# Straw return and organic fertilization promote soil organic carbon sequestration through microbes

**DOI:** 10.3389/fmicb.2026.1858111

**Published:** 2026-08-13

**Authors:** Yu Yu, Xueqing Deng, Fuwei Wang, Suzhi Xing, Jianfei Wang

**Affiliations:** 1 College of Resource and Environment, Anhui Science and Technology University, Fengyang, China; 2 Anhui Provincial Engineering Research Centre for Fertiliser Utilisation and Cultivated Land Quality Improvement of Agricultural Waste, Fengyang, China; 3 College of Agriculture, Anhui Science and Technology University, Fengyang, China

**Keywords:** microbial mediated processes, organic fertilization, soil organic carbon, straw return, sustainable agriculture

## Abstract

Soil organic carbon (C) is a critical component of terrestrial ecosystem C pools, playing a vital role in maintaining soil fertility, supporting sustainable agricultural development, and mitigating global climate change. Therefore, a comprehensive understanding of the pathways and effects of straw return and organic fertilizer application on soil C sequestration is essential. Straw return introduces exogenous C inputs that stimulate microbial activity and promote the formation of stable organic C fractions, although its decomposition is initially constrained by nitrogen (N) limitation. In contrast, organic fertilizer provides readily available stable C sources and essential nutrients, thereby enhancing soil aggregation and C stabilization. The combined application of straw return and organic fertilizer produces significant synergistic effects on soil C sequestration. Organic fertilizer alleviates microbial N deficiency caused by the high C to N ratio (C/N) of straw, facilitating more efficient straw decomposition and nutrient release. Simultaneously, it improves the functional structure of soil microbial communities, promoting the formation of mineral-associated organic C and strengthening aggregate-protected C pools. This integrated management strategy enhances short-term nutrient cycling while contributing to long-term C sequestration, offering substantial potential for advancing sustainable agriculture and climate change mitigation. This review aims to provide a theoretical basis for addressing the microbial mechanisms underlying the synergistic effect of straw returning to the soil and organic fertilizer application.

## Introduction

In the context of global climate change, soil organic carbon (C) has emerged as a central focus of research concerning agricultural soil fertility. As the largest reservoir of organic C on the planet, soil possesses a C storage capacity of approximately 2,500 Pg (Davidson and Janssens, 2006; Fang et al., 2003; Lal, 2013; Zhang et al., 2021), with SOC serving as a key component, playing a significant role in mitigating climate change. As the fundamental process of terrestrial ecosystems, the soil C cycle encompasses two key aspects: C fixation and C metabolism (Liu et al., 2020). Even minor fluctuations in this cycle can lead to profound impacts on the global C cycle (Zhang et al., 2025). However, the dynamic changes of soil C in farmland are regulated by agricultural management practices, such as the input of exogenous nutrients and straw return, which affect the microbial processes and subsequently influence the soil C cycling processes involving microorganisms (Guggenberger et al., 1999).

China has abundant crop straw resources, with an annual output exceeding 700 million tons (Ji, 2022). As a green and efficient organic C source, straw is widely used in agricultural production due to its significant improvement of soil fertility. Straw return increases soil organic matter content, improves soil structure, enhances microbial activity, and promotes crop root development, thereby enhancing crop yield and overall soil quality (Che et al., 2024; Huang et al., 2024). Additionally, straw return also provides physical protection for soil organic C and maintains its stability (Wang N. et al., 2025). The application of organic fertilizers directly inputs stable humus-like organic C into the soil and provides balanced nutrients, regulates microbial metabolic processes, and significantly enhances soil C sequestration capacity. The synergistic effect of straw return and organic fertilizer application can alleviate the adverse effects of long-term cultivation on soil structure, reduce soil erosion resulting from unreasonable cultivation practices, lower the rate of soil organic C mineralization, and improve the sequestration efficiency of soil organic C (Wang et al., 2024). Straw incorporation and organic fertilizer application are essential agricultural practices for regulating soil C sequestration capacity. By mediating microbial activities, these practices promote the formation and stabilization of soil macroaggregates, regulate the soil C-to-N ratio (C/N) and priming effect, thereby effectively enhancing soil organic C sequestration (Guo et al., 2023; Sun and Zhang, 2023; Shi et al., 2023; Wang Y. et al., 2025; Zhang et al., 2020). Existing studies demonstrated that straw incorporation combined with organic fertilizer application significantly increased the content of soil organic C and its active fractions, leading to improved soil C sequestration capacity. Long-term experiments further confirm that, compared with the application of chemical fertilizer, the combined application of straw incorporation and organic fertilizer significantly increased soil total nitrogen (N) content, microbial biomass C and N content, and crop yield. Additionally, the combined application of straw and N fertilizer facilitated the sequestration of new C in aggregates and improved C pool stability (Xi et al., 2023; Zhang et al., 2023). Therefore, the rational combined application of straw returning and organic fertilizer plays a critical role in improving soil C sequestration efficiency and promoting sustainable agricultural development.

Although there have been numerous studies separately addressing the impact of straw returning or the application of organic fertilizers on soil organic C, there remain relatively few studies on the microbial pathways of soil organic C sequestration under their combined effect. Therefore, this review aims to provide a theoretical basis for addressing the microbial pathways of soil organic C sequestration underlying the synergistic effect of straw returning and organic fertilization.

## Factors affecting microbial decomposition of straw

**Figure 1:**
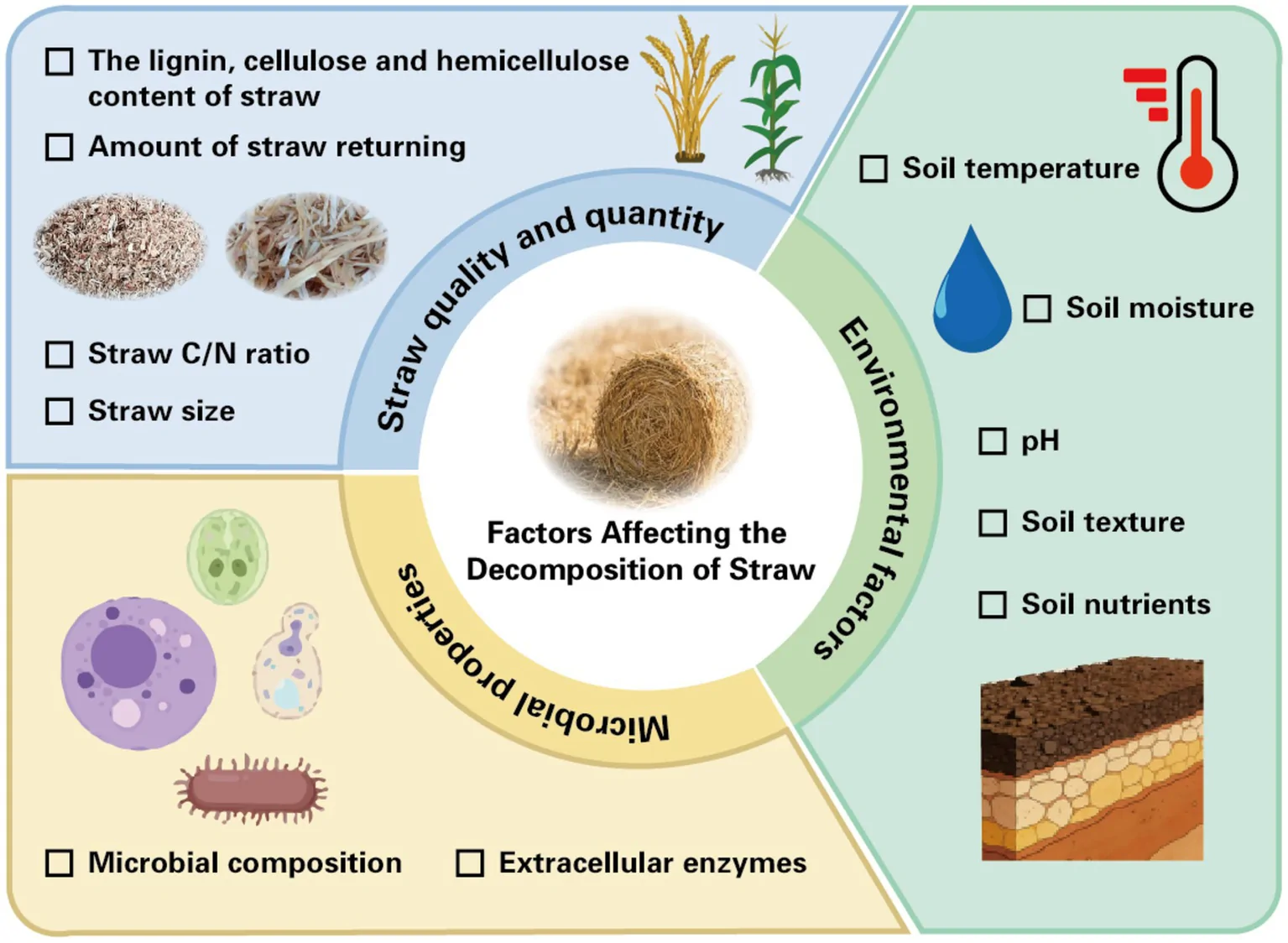
Factors affecting microbial decomposition of straw.

Straw return is a crucial strategy for enhancing soil organic matter content, facilitating nutrient cycling, and maintaining the stability of the agricultural ecosystem (Lou et al., 2011; Rui et al., 2009). The decomposition rate of crop residue is determined not only by their quality and composition, but also by the combined regulation of the environmental factors and soil microbial activity (Figure 1).

The physical and chemical properties of the crop residue itself are the main internal factors affecting its decomposition rate. Among them, the C/N is one of the most critical indicators. The optimal C/N for microbial decomposition is approximately 25:1, while the C/N of corn residue is as high as 35–46 (Feng et al., 2015; Zhang et al., 2019; Feng et al., 2015). The inherent high C/N ratio of straw stimulates microorganisms to mineralize soil organic matter to obtain N nutrients to balance their stoichiometric ratio, resulting in a shortage of available N in the soil. This competition for N between microorganisms and crops restricts efficient straw decomposition. However, the C/N of soybean residue is 14.65, which is much lower than that of corn residue and closer to the suitable range for microorganisms, but its quality loss rate during the initial stage of decomposition is actually lower than that of corn residue (Zhang et al., 2014). This phenomenon may be related to the moisture content and physical structure of the residue. Studies have shown that compared to dried and ground crop residues, fresh crop residues with high moisture content have a larger mass, larger volume, and smaller specific surface area, requiring more energy for decomposition, thereby inhibiting the decomposition by microorganisms (Hassink, 1997).

Environmental factors indirectly affect the decomposition rate by regulating the living environment of microorganisms, mainly including soil nutrients, temperature, moisture, soil texture, and pH (Fierer and Jackson, 2006; Wu et al., 2009). Soil nutrients significantly influence the straw decomposition rate. For example, soil organic matter and alkali-hydrolyzable N exhibit significant positive correlations with the straw decomposition residue rate, whereas available potassium shows a significant negative correlation (Zhang et al., 2019). Temperature is a key factor affecting the decomposition rate, as it directly affects the activity of intracellular enzymes in microorganisms. Within a certain range, an increase in temperature can significantly enhance microbial metabolism and accelerate the mineralization of crop residues. When the temperature is above 25 °C, the accumulation effect of soil organic C is significantly better than that under low-temperature conditions, indicating that temperature has a certain promoting effect on C transformation (Han J. et al., 2024). Water is also an important factor affecting the decomposition of crop residues. Appropriate moisture content can ensure the growth of aerobic microorganisms and the decomposition of crop residues. For example, the decomposition rate of crop residues in areas with higher precipitation is faster than that in areas with lower precipitation in an African forest (Tian et al., 2007). Another study further confirmed that excessive or insufficient field moisture content is not conducive to the decomposition of crop residues (Zhou et al., 2015). There are also differences in the decomposition rate of crop residues in different soil textures. Studies have shown that in soils with high clay content, the decomposition rate of crop residues is slower, which may be due to the change in the physical and biological environment of crop residue after the combination of soil clay and organic matter (Yadvinder et al., 2010; Yang et al., 2015). Soil pH affects decomposition efficiency by shaping specific microbial community structures. A previous study showed that due to the differences in physical and chemical properties, brown soil with a higher pH and sandy loam black soil with a lower pH, respectively, enriched specific bacterial and fungal groups and activated different extracellular enzyme activities, driving two different pathways of C, N, and nutrient transformation of crop residues (Wang and Cui, 2025).

The straw returning method directly affects the spatial distribution of the crop residues in the soil and the decomposition process. Previous studies showed that compared with no-tillage cover, deep plowing and returning (20–30 cm) improved soil permeability, promoted the mixing of crop residues with soil microorganisms, and significantly enhanced the proliferation of root-associated functional bacterial communities (Liu L. et al., 2023; Xu et al., 2018).

Both straw quality and environmental factors can affect soil microbial composition and activity, thereby affecting straw decomposition. Soil microorganisms drive straw decomposition through the secretion of extracellular enzymes, including cellulases, hemicellulases, and ligninase (Somchanh et al., 2026). Microbial assimilation of labile organic C via intracellular turnover contributes directly to biomass synthesis. Upon cell death, microbial necromass accumulates in soil and serves as a critical precursor for stable soil organic C, a process conceptualized as the “microbial C pump” (Liang et al., 2017). Critically, this pump operates synergistically with physical protection mechanisms. Microbial residues become occluded within soil aggregates and adsorbed onto mineral surfaces, where they undergo chemical stabilization, termed the “sequential burial effect.” This dual protection significantly extends C residence time and enhances long-term SOC persistence. Straw incorporation induces persistent shifts in the taxonomic composition and functional diversity of bacterial, fungal, and actinobacterial communities (Liang et al., 2017).

## Factors affecting the mineralisation of organic fertilisers

The mineralization of organic fertilizers is governed by a suite of interdependent biogeochemical and management-related factors. Key determinants include intrinsic fertilizer properties, particularly the C/N, as well as edaphic conditions (soil pH, texture, moisture status, and temperature) and agronomic practices. The C/N ratio serves as a pivotal biochemical threshold regulating net N dynamics. The C/N ratio below 15 typically favors net N mineralization, ratios above 20 tend to induce net N immobilization, and intermediate values (15**–**20) often support concurrent mineralization and immobilization. Critically, the magnitude of net N mineralization exhibits a significant negative correlation with the C/N ratio (Li and Li, 2012; Zhou et al., 2012). Soil pH exerts strong control over microbial metabolism: acidic conditions (pH < 5.5), characterized by elevated H^+^ and Al^3+^ concentrations, suppress microbial activity and can reduce mineralization rates by over 50%, whereas neutral to alkaline conditions (pH 6.5–8.0) generally enhance enzymatic efficiency and promote N release (Garzón et al., 2011). Temperature acts as a fundamental kinetic driver, and the mineralization rates increase exponentially within the biologically relevant range of 4–40 °C, with peak nitrification activity observed near 28 °C, thereby optimizing plant-available N supply (Cannavo et al., 2022). Soil moisture modulates mineralization primarily through its influence on oxygen diffusion and substrate accessibility. Optimal mineralization occurs at soil water-filled pore space (WFPS) levels of 50–70%. Both waterlogging (WFPS > 80%) and severe drought (WFPS < 30%) constrain aerobic microbial processes and impede mineralization (Cannavo et al., 2022; De Jesus et al., 2024). Soil texture can also affect organic fertilizer mineralization. High clay content may limit net N mineralization via physical protection of organic matter and reduced microbial mobility (Li and Li, 2012). Furthermore, co-application with inorganic fertilizers, especially in low-fertility soils, can effectively lower the apparent C/N ratio, stimulate microbial biomass and activity, and thereby accelerate mineralization. The C/N ratio and pH constitute primary regulatory levers, whereas temperature and moisture act as rate-controlling environmental modulators, exerting their influence predominantly through differential regulation of microbial community structure and metabolic activity.

## Effects of straw return on soil organic C sequestration

Straw returning to the soil is a key measure to enhance the soil C sequestration capacity, which not only effectively avoids resource waste but also helps to increase soil fertility and promote the agricultural sustainability and environmental protection of the crop system (Qasim et al., 2022); however, in practical applications, there may be a problem of short-term nutrient depletion (Li et al., 2024).

Straw is mainly composed of cellulose, hemicellulose, lignin, and a small amount of proteins, sugars, and elements such as N, phosphorus, and potassium. Studies have shown that applying straw as an external C source to the soil is an effective measure to increase the organic C storage in agricultural ecosystems (Liu S. et al., 2025; Tang et al., 2024). The core mechanism lies in that straw, as an external organic C input, changes the soil microbial community and its living environment, driving the C transformation and storage process dominated by microorganisms (Liu Y. et al., 2025; Zhang M. et al., 2024). Research shows that the input of straw directly provides a rich C source for soil microorganisms, thereby stimulating the activity and abundance of the microbial community (Yu et al., 2020). Moreover, it affects the soil chemical properties, microbial activity and diversity, as well as the community structure and functional diversity of the community, thereby influencing the transformation of soil organic C (Che et al., 2024; Zhang J. et al., 2024). In the rice-wheat rotation system, long-term straw cover returning can significantly change the physical and chemical properties of the soil, creating a more suitable environment for microorganisms, thereby promoting their decomposition and transformation of straw, accelerating the accumulation of organic C (Yang et al., 2020). At the same time, the addition of straw will change the chemical composition of soil organic C, especially promoting the formation of particulate organic C and mineral-bound organic C, which is closely related to the adjustment of specific microbial communities (Li et al., 2021). Long-term straw returning not only changes the community structure of microorganisms but also significantly enhances the functionality of the microbial community, including the increase in enzyme activity related to the C cycle and the complexity of the microbial interaction network, which further strengthens the soil ability to hold organic C, forming a positive feedback loop of continuous C accumulation (Yang et al., 2021). Additionally, straw returning measures have a positive synergistic effect on soil organic C stability and crop yield by influencing soil nutrients, aggregation, and the ease of decomposition of C (Huang et al., 2021; Wang et al., 2026).

Although straw returning has multiple potential benefits, it may cause short-term nutrient depletion in the soil during the initial stage of decomposition. This is because the C/N of straw is relatively high, and during the decomposition of straw by microorganisms, they will retain the available N in the soil to meet their own metabolic needs, resulting in competition between microorganisms and crops for N, which may lead to insufficient nutrient supply for crops in the early growth stage (Lv et al., 2023; Mo et al., 2024). Therefore, a reasonable straw returning mode requires the application of N fertilizers or other nutrient management strategies to balance the nutrient supply in the soil (Huang et al., 2024; Zhao et al., 2024).

## Effects of organic fertilizer on soil organic C sequestration

Organic fertilizer, as an important external C input in the agricultural ecosystem, plays a crucial role in maintaining soil productivity and the balance of C cycling. On the one hand, it provides external organic matter, stimulating the activity of soil microorganisms, promoting the decomposition, transformation, and humification of organic matter, thereby facilitating the conversion of active organic C into stable SOC (Luo et al., 2017; Tang and Hu, 2025; Zhou et al., 2024); on the other hand, the input of external organic matter can promote the formation of large particle aggregates, enhancing the soil C sequestration capacity through physical protection (Fang, 2021; Ten Damme et al., 2020). Organic fertilizer increased the SOC content by 29.6–119.8% (0–15 cm soil layer) and 10.3–36.3% (15–30 cm soil layer), respectively, indicating that the C sequestration effect of organic fertilizer is mainly concentrated in the surface layer (Li S. et al. 2023). And its positive impact on deep soil gradually becomes apparent with the increase in fertilization years. Another study further confirmed that long-term organic fertilizer return can effectively increase the content of particulate organic C, soluble organic C, and active organic C in the soil, thereby significantly enhancing SOC storage (Pei et al., 2023). This positive effect continues to strengthen with the extension of fertilization years. In addition, organic fertilizer can also meet the nutritional needs of crops for macronutrients such as N, phosphorus, and potassium, as well as various micronutrients, through the mechanism of slow-release nutrients, thereby reducing environmental pollution (Allahverdiyev and Khalilov, 2021; Badu Brempong and Addo-Danso, 2022; Yue et al., 2023). Therefore, the application of organic fertilizer maintains the nutrient balance of the soil-crop system while synergistically enhancing the soil C sink capacity and environmental benefits (Han C. et al., 2024; Liu et al., 2025).

## Synergistic C sequestration mechanism of straw returning and organic fertilizer application

**Figure 2:**
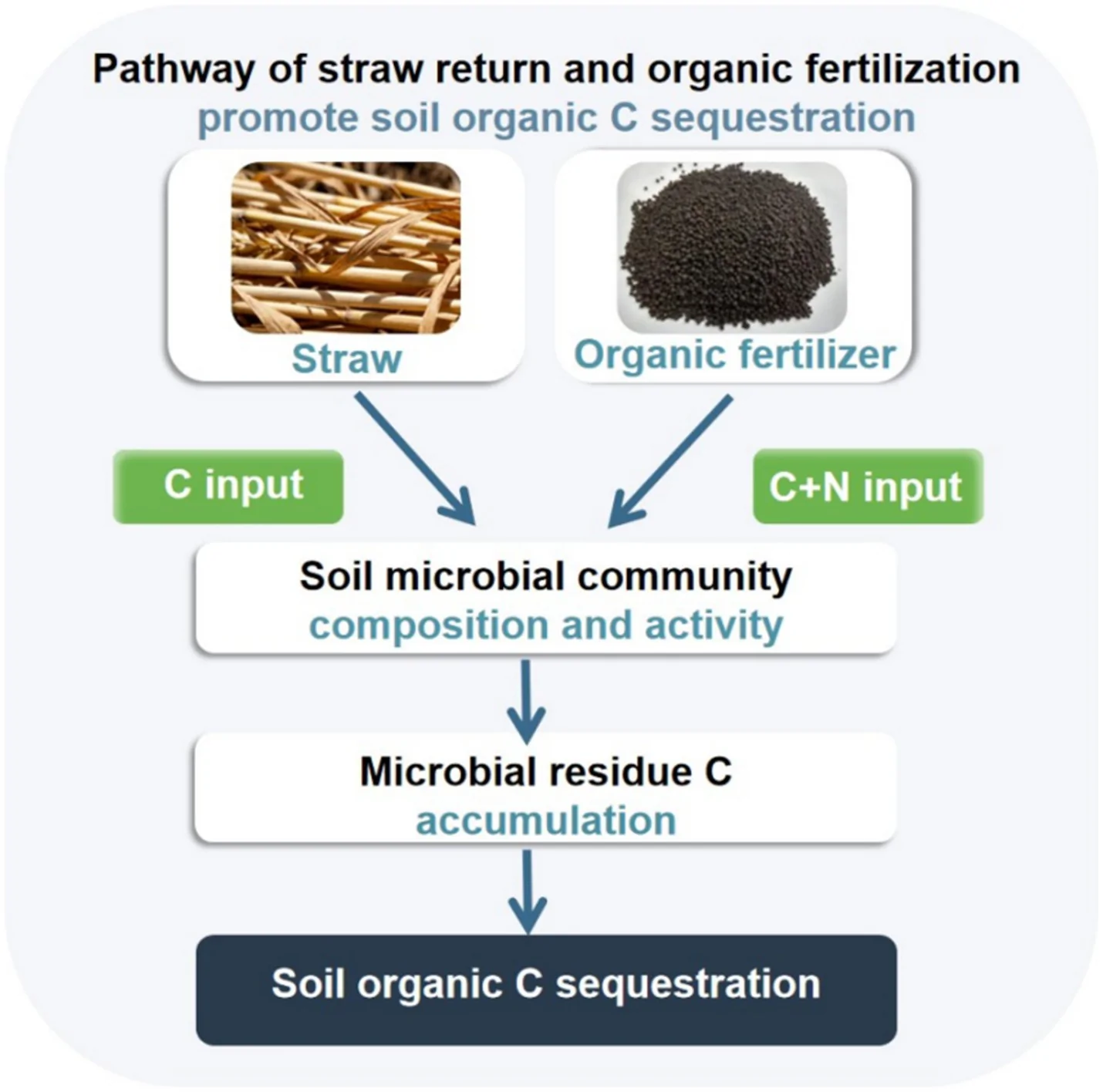
The *C* sequestration pathway of the combined application of straw and organic fertilizer through microbes.

Straw returning to the soil and the combined application of organic fertilizers are important research directions in the field of agricultural sustainable development. Their main advantages lie in the complementary effect of the two, which jointly enhances soil fertility and C sequestration capacity (Hu et al., 2023; Liu X. et al., 2023). When only straw is returned to the soil, there is usually a C-N imbalance problem, lacking sufficient N to support microorganisms to reach the optimal growth state (Mao et al., 2024). Organic fertilizers are rich in N and various nutrients necessary for microorganisms, and their addition can effectively alleviate the microbial N limitation caused by the excessively high C/N in straw alone, providing sufficient N sources for microorganisms and driving their enzymatic hydrolysis process of straw (Figure 2). Under the condition of guaranteed N sources, microorganisms can more efficiently secrete key extracellular enzymes such as cellulose-degrading enzymes, achieving rapid decomposition of stubborn organic components in straw (Li S. et al. 2023; Song et al., 2024; Yan et al., 2026). The rational combination of straw and organic fertilizers has a synergistic improvement effect on soil structure and microbial communities. On the one hand, the combined application of the two can promote the formation of soil aggregates, thereby improving soil structure, enhancing soil water-holding and nutrient retention capacity and aeration, and creating favorable conditions for root growth (Ma et al., 2024; Wen et al., 2024; Xu et al., 2026); on the other hand, the interaction between aggregate formation and microbial activity optimizes the composition and functional diversity of the microbial community, enhances metabolic activity, and provides a stable and balanced C source and living environment for microorganisms (Huang et al., 2025; Li et al., 2025; Mehran et al., 2025; Wen et al., 2025). In conclusion, the combined application of straw and organic fertilizers enhances soil C sequestration capacity through multiple mechanisms such as optimizing nutrient cycling, improving the diversity and metabolic function of soil microorganisms, promoting aggregate formation, etc. This combined application mode provides an effective path for improving the C sink function of farmland soil and achieving agricultural sustainable development.

## Conclusion

This article systematically explores the regulatory effects and mechanisms of agricultural management measures on soil C sequestration capacity from three perspectives: straw returning to the soil, application of organic fertilizers, and their combined application. The research shows that straw returning to the soil and the application of organic fertilizers can significantly enhance soil C sequestration capacity by increasing soil organic C content, improving soil structure, activating microbial activity, and promoting the physical protection of organic C by aggregates. However, the synergistic C sequestration mechanism of straw returning and organic fertilizer application, as well as the key regulatory links in the microbial metabolic process, still need to be further analyzed to provide a more precise theoretical basis and technical support for optimizing agricultural management measures and achieving the synergy of C sequestration and C sink enhancement while achieving high crop yields.
